# Feasibility of a multicentre, randomised controlled trial of laparoscopic versus open colorectal surgery in the acute setting: the LaCeS feasibility trial protocol

**DOI:** 10.1136/bmjopen-2017-018618

**Published:** 2018-02-22

**Authors:** Deena Harji, Helen Marshall, Katie Gordon, Hannah Crow, Victoria Hiley, Dermot Burke, Ben Griffiths, Catherine Moriarty, Maureen Twiddy, John L O’Dwyer, Azmina Verjee, Julia Brown, Peter Sagar

**Affiliations:** 1 Newcastle Centre for Bowel Disease, Royal Victoria Infirmary, Newcastle Upon Tyne, UK; 2 Clinical Trials Research Unit, University of Leeds, Leeds, UK; 3 John Goligher Colorectal Department, St James' University Hospital, Leeds, UK; 4 Leeds Institute of Health Sciences, University of Leeds, Leeds, UK; 5 Academic Unit of Health Economics, Leeds Institute of Health Sciences, University of Leeds, Leeds, UK; 6 Patient and Public Representative, Association of Coloprocotology of Great Britain and Ireland, London, UK

**Keywords:** colorectal surgery, clinical trials, adult surgery

## Abstract

**Introduction:**

Acute colorectal surgery forms a significant proportion of emergency admissions within the National Health Service. There is limited evidence to suggest minimally invasive surgery may be associated with improved clinical outcomes in this cohort of patients. Consequently, there is a need to assess the clinical effectiveness and cost-effectiveness of laparoscopic surgery in the acute colorectal setting. However, emergency colorectal surgical trials have previously been difficult to conduct due to issues surrounding recruitment and equipoise. The LaCeS (randomised controlled trial of Laparoscopic versus open Colorectal Surgery in the acute setting) feasibility trial will determine the feasibility of conducting a definitive, phase III trial of laparoscopic versus open acute colorectal resection.

**Methods and analysis:**

The LaCeS feasibility trial is a prospective, multicentre, single-blinded, parallel group, pragmatic randomised controlled feasibility trial. Patients will be randomised on a 1:1 basis to receive either

laparoscopic or open surgery. The trial aims to recruit at least 66 patients from five acute general surgical units across the UK. Patients over the age of 18 with a diagnosis of acute colorectal pathology requiring resection on clinical and radiological/endoscopic investigations, with a National Confidential Enquiry into Patient Outcome and Death classification of urgent will be considered eligible for participation. The primary outcome is recruitment. Secondary outcomes include assessing the safety profile of laparoscopic surgery using intraoperative and postoperative complication rates, conversion rates and patient-safety indicators as surrogate markers. Clinical and patient-reported outcomes will also be reported. The trial will contain an embedded qualitative study to assess clinician and patient acceptability of trial processes.

**Ethics and dissemination:**

The LaCeS feasibility trial is approved by the Yorkshire and The Humber, Bradford Leeds Research Ethics Committee (REC reference: 15/ YH/0542). The results from the trial will be presented at national and international colorectal conferences and will be submitted for publication to peer-reviewed journals.

**Trial registration number:**

ISRCTN15681041; Pre-results.

**Strengths and limitations of this study**

- This trial will assess the feasibility and acceptability of conducting a definitive, phase III randomised controlled trial of laparoscopic versus open emergency colorectal resection.
- The main challenges regarding recruitment, randomisation, equipoise, blinding and follow-up will be identified through the use of an embedded qualitative study.
- The main limitations of this trial are the lack of power to examine efficacy.

## Background

Emergency general surgery is a huge clinical service, with approximately 1000 finished consultant episodes per 100 000 population/year.^1 2^ Approximately 30% of emergency admissions are secondary to colorectal pathology, namely, colorectal malignancy, inflammatory bowel disease and diverticular disease.^3 4^ More than 30 000 patients undergo an emergency laparotomy for a variety of intra-abdominal pathologies each year within the National Health Service (NHS) in England and Wales.^5^ In the UK, the National Emergency Laparotomy Audit (NELA) reported outcomes on 23 198 patients, of which 37% underwent an emergency colorectal resection between December 2014 and November 2015.^6^ The burden of emergency surgery is significant, with reports of 30-day postoperative morbidity rates of 33%–71% and mortality rates of 14%–17%.

The NELA audit reports that the majority of emergency surgery is undertaken using an open approach, with approximately 14% of all emergency abdominal operations commenced laparoscopically, of which only half are completed laparoscopically.^6^ The role of laparoscopic surgery in certain acute intra-abdominal pathologies (ie, acute appendicitis) has been well elucidated in a number of randomised controlled trials, with reports of improved pain, shorter recovery and reduced length of hospital stay.^7 8^ Consequently, laparoscopic appendicectomy has become a well-established technique.^9^ In comparison, the current evidence of acute laparoscopic colorectal resection consists of a number of case series and cohort studies, which are limited by their retrospective nature, strict patient selection and small sample size.^10 11^ The evidence base for laparoscopic surgery in the elective colorectal surgery is vast.^12 13^ However, applying this evidence to the acute setting is inappropriate due to the varying levels of sepsis, differing patient physiology and potentially more advanced disease states. The only way to integrate laparoscopic surgery in the algorithm for acute colorectal pathology is to evaluate its safety and efficacy within the remit of a randomised controlled trial.

Surgical trials have been traditionally deemed to be difficult to undertake due to a range of practical and methodological challenges, including difficulties in recruitment, randomisation and lack of surgical equipoise.^14^ These issues are further amplified in the emergency setting and therefore, it is important to conduct a feasibility trial to assess key trial processes to ensure successful delivery of a future definitive trial. This protocol paper outlines the LaCeS (Laparoscopic versus open Colorectal Surgery in the acute setting feasibility trial). The trial aims to assess the feasibility, safety and acceptability of performing a large-scale definitive phase III randomised controlled trial comparing emergency laparoscopic surgery with open surgery for acute colorectal pathology.

## Methods

### Design

The LaCeS feasibility trial is a prospective, multicentre, single-blinded, parallel group, pragmatic randomised controlled feasibility trial. At least 66 participants will be randomised on an equal basis to receive either laparoscopic surgery or open surgery across five UK centres.

**Figure 1:**
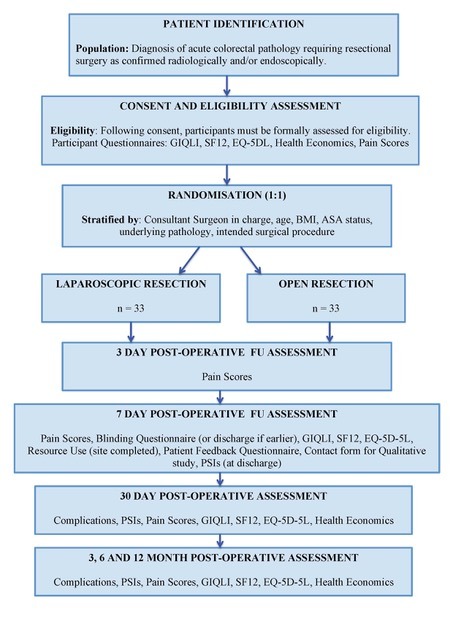
Trial schema.

Participants will be blinded to the randomisation allocation until 7 days after surgery or the day of discharge, if earlier. Participants will be followed up at prespecified time intervals; 3 days, 7 days, 30 days, 3 months and 6 months postoperatively. In addition, some patients will also be followed up 12 months postoperatively to assess the feasibility of collecting data out to this time point. The trial schema is outlined in figure 1.

## Primary outcome

The primary outcome measure is recruitment. The trial aims to recruit at least 66 patients over a 15-month period across five UK centres, with a steady state of recruitment of five patients per month over the last 12 months of the trial period.

## Secondary outcomes

Key secondary outcomes include:

- To pilot the recruitment and randomisation processes and assess their acceptability to clinicians and patients within the emergency setting;
- To assess the safety profile of emergency laparoscopic surgery;
- To explore the potential optimal endpoints, either clinical or patient-reported, that could be used as a primary endpoint in a definitive phase III trial;
- To explore the practical application and success of blinding in the emergency setting;
- To test the feasibility and refine the strategy for collecting patient-reported quality-of-life data and resource-use data to inform a future economic evaluation.

## Study population

The study population is those presenting to emergency general surgery services with an acute colorectal pathology requiring urgent resectional surgery.

## Setting

The study is being undertaken in five NHS hospitals with acute general surgery services, able to deliver emergency laparoscopic surgery. These hospitals are a mixture of teaching hospitals and district general hospitals with dedicated emergency surgery services.

## Eligibility

### Patient inclusion criteria

- Aged ≥18 years.
- Diagnosis of acute colorectal pathology requiring resectional surgery (eg, acute diverticular disease, inflammatory bowel disease and colorectal cancer) confirmed radiologically and/or endoscopically.
- National Confidential Enquiry into Patient Outcome and Death classification of urgent.^15^
  - Defined as intervention for acute onset or clinical deterioration of potentially life-threatening conditions, for those conditions that may threaten the survival of limb or organ. Normally within hours of decision to operate, subdivided into NELA categories of 2a (~2–6 hours) or 2b (~6–18 hours).
- Suitable candidate for surgery as judged by the operating surgeon.
- Suitable for laparoscopic and open surgery in the opinion of the operating surgeon.
- Suitable for laparoscopic and open surgery in the opinion of the anaesthetist.
- Informed written consent obtained.
  - In cases where the patient’s judgement is considered temporarily impaired in relation to the condition causing his/her admission, for example, experiencing significant pain/distress/nausea or acute delirium secondary to sepsis, personal consultee advice would be appropriate.

### Patient exclusion criteria

- Haemodynamic instability requiring inotropic support.
- Acute non-colorectal pathology (eg, adhesional small bowel obstruction, appendicitis, peptic ulcer disease, etc).
- Hand-assisted laparoscopic surgery.
- Laparoscopy and peritoneal lavage alone for colorectal pathology.
- Insertion of an endoscopic stent followed by laparoscopic resection for colorectal pathology.
- Patients undergoing surgery for complications of elective colorectal operations.
- Pregnancy.
- Pre-existing cognitive impairment.
- Currently participating in another surgical trial.

### Site eligibility

The trial will be performed as a multicentre collaboration within the UK across approximately five sites. Participation of sites will be dependent on the following criteria:

- Has dedicated emergency surgery services with appropriate provisions for emergency laparoscopic surgery;
- Has dedicated elective laparoscopic colorectal surgery services;
- Established previous involvement in clinical trials;
- Anticipating to recruit at least two to three patients per month.

### Surgeon eligibility

All participating consultant surgeons must have a subspecialist interest in colorectal surgery and have performed a minimum of 50 laparoscopic colorectal resections and must perform at least 20 laparoscopic resections a year, with equivalent experience with open surgery (this can include procedures in both the emergency and elective setting).

## Recruitment and randomisation process

All patients with suspected acute colorectal pathology will be assessed clinically, radiologically and/or endoscopically as per best clinical practice. Following confirmation of clinical and radiological/endoscopic diagnosis of an acute colorectal pathology requiring resection, patients will be approached for participation in the trial. Patients will only be approached for potential participation between the hours of 08:00 – 22:00.

Patients who are deemed to have temporary impairment in their judgement (temporary lack of mental capacity), related to their condition (eg, experiencing significant pain/distress/nausea or acute delirium secondary to sepsis), can be entered into the trial if a personal consultee can be identified to advise about trial entry. A personal consultee will ideally be a family member or partner, and will be informed of all key trial processes. Once the patient regains capacity, written informed consent will be requested from the patient for ongoing participation within the trial. Given the emergency nature of the trial, the time available to consider participation will be shorter than in the elective setting. Patients and personal consultees will be given as long as they need to consider participation in the trial. Ideally, this will be at least 2 hours.

Following appropriate surgical and anaesthetic assessment and confirmation of the clinical diagnosis, patients will be randomised using a telephone or online randomisation system, on a 1:1 basis to receive either laparoscopic or open surgery. Patients will be stratified to one of the two arms according to intended consultant surgeon in charge, age, body mass index, American Society of Anesthesiologists status, nature of underlying pathology and intended surgical procedure.

## Trial interventions

### Surgery

For the purposes of this pragmatic trial, surgery, either open or laparoscopic, will be undertaken in accordance with local standard practice. Laparoscopic surgery includes the use of multiport and single-port incisions to establish pneumoperitoneum to enable surgical resection. Conversion to an open operation is defined as the use of a midline laparotomy wound for any part of the colorectal dissection. The use of a limited laparotomy wound to facilitate specimen extraction is permissible.

### Blinding

The process of blinding this patient population within the emergency setting will be piloted in this feasibility study. Participants will be blinded to the randomisation allocation for 7 days postoperatively or until the day of discharge, if earlier. Hypoallergenic dressings will be applied to mimic the distribution of the midline laparotomy wound and lateral port site wounds. To assess the success of the blinding protocol, the Bang Blinding Index will be used to calculate the proportion of non-blinded participants in the trial on day 7 postoperatively.^16^

## Outcome assessment

### Primary outcome: recruitment

The primary outcome of this trial is recruitment. Data logs will be kept to assess:

- the number of patients screened for eligibility,
- the proportion of eligible patients consenting to participation and reasons for non-participation,
- the proportion of consenting patients undergoing randomisation and reasons for non-randomisation,
- the proportion of patients not receiving their randomised allocation and the reasons for this.
- The combination of quantitative and qualitative data regarding recruitment will enable us to understand the potential pool of eligible patients and reasons for non-participation and withdrawal throughout the recruitment process. This will enable us to further refine and develop our recruitment and randomisation processes for a definitive, phase III trial.

### Secondary outcomes

#### Safety

To assess the safety profile of acute laparoscopic surgery, the following outcomes will be assessed: conversion rates from laparoscopic surgery to open surgery, intraoperative and postoperative complication rates, the severity of postoperative complications using the Clavien-Dindo grading system, the incidence of patient-safety indicators and 30-day postoperative mortality rates.

#### Endpoint evaluation to identify the optimal primary endpoint(s) for a definitive phase III trial

A range of key outcomes will be collected, including:

- Clinical outcomes including length of high dependency unit/intensive care unit stay, length of hospital stay, resumption of gastrointestinal function and oral intake, opioid analgesic use, reoperation rates and readmission rates and details regarding histopathology of the resected specimen;
- Patient-reported health-related quality-of-life data using the Gastrointestinal Quality of Life Index, the Short Form - 12 Health Survey, pain scores using the Brief Pain Inventory and the EuroQol-5D-5L;
- Resource use using dedicated patient-reported and site-completed Health Economics Questionnaires to measure primary and secondary healthcare service use;
- Patient and clinician acceptability of trial processes and procedures using in-depth qualitative interviews and a dedicated Patient Feedback Questionnaire.

These candidate endpoints will be explored quantitatively and qualitatively to assess for completion rates, generate data to inform future power calculations and to identify which endpoint will be of most meaning and value to clinicians and patients as a primary endpoint for a definitive phase III trial. Candidate endpoints will be collected at various times during the course of the trial (table 1). Trial follow-up will cease when the last participant reaches 6 months postrandomisation.

**Table 1:** Schedule of events

	Pretrial diagnostics	Baseline	Operative	3-Day postoperative review	7-Day postoperative review	30-Day postoperative review	3, 6 and 12* months postoperative assessment
Radiological/endoscopic diagnosis	✓						
Medical assessment		✓		✓	✓	✓	✓
Participant completed questionnaires		✓		✓	✓	✓	✓
Operative details			✓				
Complications			✓			✓	✓
Patient-safety indicators					✓ At discharge		✓†
Patient Feedback Questionnaire					✓		
Blinding Questionnaire					✓		
Resource usage			✓		✓ At discharge		

*Trial follow-up will cease when the last participant reaches 6 months postrandomisation.

†In-patients only

## Qualitative substudy

Trial processes and their acceptability to clinicians and patients will be assessed using semistructured, in-depth qualitative interviews to optimise and design strategies for a definitive, phase III trial. Clinicians will be interviewed regarding overall trial processes, recruitment in the emergency setting and potential primary endpoints for a future phase III trial. Patients will be interviewed to identify any issues with the randomisation process, preferential bias for one type of surgery, reasons for non-participation or withdrawal, refusal of treatment allocation and burden of participation.

## Sample size calculation and statistical analysis

The sample size has been chosen to allow the estimation of the parameters of interest to the necessary degree of precision, following the recommended rule-of-thumb of 30 participants per arm.^17^ The sample size has been calculated to account for a 10% attrition rate and aims to recruit at least 66 patients. This sample size will allow the estimation of morbidity and mortality rates with the laparoscopic arm with 95% two-sided CIs of at most ±17%, allowing its safety profile to be demonstrated. Achievement of this recruitment target will also demonstrate feasibility of a likely required recruitment rate for a successful, definitive, phase III trial.

The feasibility of recruitment and randomisation will be evaluated by summarising the screening, eligibility, consent and randomisation processes, including numbers of participants involved during each stage. Descriptive summaries of the participant recruitment pathways at the five recruiting centres will be presented. Reasons for non-participation in the study will be summarised. Participant retention during follow-up, including number of participants completing/withdrawing from the study and reasons for withdrawal, will be presented by treatment arm. Completion rates of data collected at the baseline and follow-up visits will be summarised. The Bang Blinding Index at 7 days and the timings of non-blindings will be reported to inform the feasibility of blinding in a phase III trial. In addition, the relationship between patients, surgical team members and centres will be described to indicate the clustering structure of the feasibility study to inform the design of a phase III trial. The safety profile of each treatment arm will be summarised through descriptive statistics. Mortality rates, intraoperative and postoperative complication rates, conversion rates and patient-safety indicator rates will be reported with 95% CIs. All analyses will be conducted on an intention-to-treat basis. An analysis formally comparing the two treatment arms will not be performed due to the lack of power within this feasibility study, in addition to the purpose of this study.

## Ethics

The trial will be performed in accordance with the principles of good clinical practice in clinical trials and the recommendations guiding physicians in biomedical research involving human subjects adopted by the 18th World Medical Assembly, Helsinki, Finland, 1964, amended at the 64th World Medical Association General Assembly, Fortaleza, Brazil, October 2013. Informed written consent will be obtained from the participants (or from personal consultees where appropriate) prior to randomisation into the study. The right of a patient to refuse participation without giving reasons will be respected. Participants remain free to withdraw at any time from the study without giving reasons and without prejudicing his/her further treatment.

## Dissemination

The results of this trial will be presented at relevant colorectal scientific meetings and will be published in peer-reviewed journals.

## Discussion

There is a lack of high-quality evidence on laparoscopic surgery for emergency colorectal resection. There are a number of well-documented challenges in undertaking emergency surgery trials, including issues with recruitment, safety and surgical equipoise.^18–20^ The LaCeS feasibility trial is a necessary requirement prior to embarking on a definitive, phase III trial. Conducting this feasibility trial with an embedded qualitative study will enable a greater understanding of trial processes and their acceptability, thus allowing refinement of methodology and infrastructure for a planned, robust, definitive trial.

This feasibility trial is the first of its kind to assess the role of resectional laparoscopic surgery in the acute colorectal setting. The trial aims to assess the role of blinding in the acute clinical scenario, the inclusion of patients with temporary loss of capacity and aims to determine the barriers to recruitment and participation within this framework. The evidence generated from this trial will not only help inform the design of a definitive, phase III trial but will also help inform future methodological work in recruiting and randomising patients in the emergency setting. Emergency surgery research, and in particular acute colorectal surgery research, has been limited to individual case series and cohort studies, due to perceived difficulties in recruitment, randomisation and retention of patients. The LaCeS feasibility trial will try to understand these issues and offer solutions to help overcome them through consultation with participating surgeons, patients, the trial management group and the trial steering committee. This will lead to the design of a pragmatic, phase III trial, which will reflect the opinions of all key stakeholders.

## Supplementary Material

Reviewer comments

Author's manuscript
