# Supplementary material for: Feasibility of a multicentre, randomised controlled trial of laparoscopic versus open colorectal surgery in the acute setting: the LaCeS feasibility trial protocol
Source: BMJ Open. 2018 Feb 22;8(2):e018618. doi: 10.1136/bmjopen-2017-018618 (PMC5879497; doi:10.1136/bmjopen-2017-018618)
Supplement: Author's manuscript [file bmjopen-2017-018618.draft_revisions.pdf]

BMJ Open is committed to open peer review. As part of this commitment we make the peer review history of every article we publish publicly available.

When an article is published we post the peer reviewers' comments and the authors' responses online. We also post the versions of the paper that were used during peer review. These are the versions that the peer review comments apply to.

The versions of the paper that follow are the versions that were submitted during the peer review process. They are not the versions of record or the final published versions. They should not be cited or distributed as the published version of this manuscript.

BMJ Open is an open access journal and the full, final, typeset and author-corrected version of record of the manuscript is available on our site with no access controls, subscription charges or pay-per-view fees (http://bmjopen.bmj.com).

If you have any questions on BMJ Open's open peer review process please email editorial.bmjopen@bmj.com

BMJ Open

The feasibility of a multi-centre, randomised controlled trial of laparoscopic versus open colorectal surgery in the acute setting – The LaCeS Feasibility Trial Protocol

Journal:	*BMJ Open*
Manuscript ID	bmjopen-2017-018618
Article Type:	Protocol
Date Submitted by the Author:	14-Jul-2017
Complete List of Authors:	Harji, Deena; Northern Deanery; University of Leeds Clinical Trials Research Unit<br>Marshall, Helen; University of Leeds Clinical Trials Research Unit<br>Gordon, Kathryn; University of Leeds Clinical Trials Research Unit, CTRU<br>Crow, Hannah; University of Leeds Clinical Trials Research Unit<br>Hiley, Victoria; University of Leeds Clinical Trials Research Unit<br>Burke, Dermot; St James' University Hospital, John Goligher Colorectal Department<br>Griffiths, Ben ; Newcastle Upon Tyne Hospitals NHS Foundation Trust<br>Moriarty, Catherine ; St James' University Hospital , John Goligher Colorectal Department<br>Twiddy, Maureen; University of Leeds, Leeds Institute of Health Sciences<br>O'Dwyer, John; University of Leeds Leeds Institute of Health Sciences, Academic Unit of Health Economics<br>Verjee, Azmina<br>Brown, Julia; University of Leeds, Leeds Institute of Clinical Trials Research<br>Sagar , Peter; St James' University Hospital, John Goligher Colorectal Department
**Primary Subject Heading**:	Surgery
Secondary Subject Heading:	Surgery
Keywords:	laparoscopic surgery, Colorectal surgery < SURGERY, feasibility study, randomised controlled trial, emergency surgery

SCHOLARONE™ Manuscripts

The feasibility of a multi-centre, randomised controlled trial of laparoscopic versus open colorectal surgery in the acute setting – The LaCeS Feasibility Trial Protocol

Corresponding Author

Miss Deena Harji

Northern Deanery, Waterfront 4, Goldcrest Way, Newburn Riverside, Newcastle Upon Tyne, NE15 8NY

Clinical Trials Research Unit, University of Leeds, Leeds

deena.harji@hotmail.com

Co-Authors

Mrs Helen Marshall

Clinical Trials Research Unit, University of Leeds, Leeds

H.C.Marshall@leeds.ac.uk

Ms Katie Gordon,

Clinical Trials Research Unit, University of Leeds, Leeds

k.a.gordon@leeds.ac.uk

Miss Hannah Crow

Clinical Trials Research Unit, University of Leeds, Leeds

h.e.crow@leeds.ac.uk

Mrs Victoria Hiley

Clinical Trials Research Unit, University of Leeds, Leeds

V.Hiley@leeds.ac.uk

Mr Dermot Burke

St James's University Hospital, Beckett Street, Leeds

D.Burke@leeds.ac.uk

Mr Ben Griffiths

For peer review only

Royal Victoria Infirmary, Queen Victoria Road, Newcastle

ben.griffiths@nuth.nhs.uk

Mrs Catherine Moriarty

St James's University Hospital, Beckett Street, Leeds

catherine.moriarty@nhs.net

Dr Maureen Twiddy

Leeds Institute of Health Sciences, University of Leeds, Leeds

M.Twiddy@leeds.ac.uk

Mr John L O'Dwyer

Academic Unit of Health Economics, Leeds Institute of Health Sciences, University of Leeds, Leeds

j.odwyer@leeds.ac.uk

Miss Azmina Verjee

Patient and Public Involvement Representative

azminaverjee@hotmail.com

Professor Julia Brown

Clinical Trials Research Unit, University of Leeds, Leeds

J.M.B.Brown@leeds.ac.uk

Professor Peter Sagar

St James's University Hospital, Beckett Street, Leeds

petersagar@aol.com

Word Count: 2622

Abstract

Introduction

For peer review only

Acute colorectal surgery forms a significant proportion of emergency admissions within the NHS. There is limited evidence to suggest minimally invasive surgery may be associated with improved clinical outcomes in this cohort of patients. Consequently, there is a need to assess the clinical effectiveness and cost-effectiveness of laparoscopic surgery in the acute colorectal setting. However, emergency colorectal surgical trials have previously been difficult to conduct due to issues surrounding recruitment and equipoise. The LaCeS (randomised controlled trial of **La**paroscopic versus open **C**olorectal **S**urgery in the acute setting) feasibility trial will determine the feasibility of conducting a definitive, phase III trial of laparoscopic versus open acute colorectal resection.

Methods and Analysis

The LaCeS feasibility trial is a prospective, multicentre, single blinded, parallel group, pragmatic randomised controlled feasibility trial. Patients will be randomised on a 1:1 basis to receive either laparoscopic or open surgery. The trial aims to recruit at least sixty-six patients from five acute general surgical units across the United Kingdom. Patients over the age of 18 with a diagnosis of acute colorectal pathology requiring resection on clinical and radiological/endoscopic investigations, with an NCEPOD classification of urgent will be considered eligible for participation. The primary outcome is recruitment. Secondary outcomes include assessing the safety profile of laparoscopic surgery using intra- and post-operative complication rates, conversion rates and patient safety indicators as surrogate markers. Clinical, patient-reported costs and outcomes will also be reported. The trial will contain an embedded qualitative study to assess clinician and patient acceptability of trial processes.

Ethics and Dissemination

The LaCeS feasibility trial is approved by the Yorkshire and The Humber, Bradford Leeds Research Ethics Committee (REC reference: 15/YH/0542). The results from the trial will be presented at national and international colorectal conferences and will be submitted for publication to peer-reviewed journals.

Strengths and Limitations

This trial will assess the feasibility and acceptability of conducting a definitive, phase III randomised controlled trial of laparoscopic versus open emergency colorectal resection.

For peer review only

The main challenges regarding recruitment, randomisation, equipoise, blinding and follow-up will be identified through the use of an embedded qualitative study.

The main limitations of this trial are the lack of power to examine efficacy.

Trial Registration

Trial registration number: ISRCTN15681041. Registered on 18th April 2016.

Keywords

Colorectal surgery – feasibility study – randomised controlled trial – emergency surgery – laparoscopic surgery

Background

Emergency general surgery is a huge clinical service, with approximately 1000 Finished Consultant Episodes per 100,000 population/year [1, 2]. Approximately 30% of emergency admissions are secondary to colorectal pathology, namely, colorectal malignancy, inflammatory bowel disease and diverticular disease [3, 4]. More than 30,000 patients undergo an emergency laparotomy for a variety of intra-abdominal pathologies each year within the NHS in England and Wales [5]. In the UK, the National Emergency Laparotomy Audit (NELA) reported outcomes on 23,198 patients, of which 37% underwent an emergency colorectal resection between December 2014 and November 2015 [6]. The burden of emergency surgery is significant, with reports of 30 day postoperative morbidity rates of 33-71% and mortality rates of 14-17%.

The NELA audit reports that the majority of emergency surgery is undertaken using an open approach, with approximately 14% of all emergency abdominal operations commenced laparoscopically, of which only half are completed laparoscopically [6]. The role of laparoscopic surgery in certain acute intra-abdominal pathologies i.e. acute appendicitis has been well elucidated in a number of randomised controlled trials, with reports of improved pain, shorter recovery and reduced length of hospital stay [7, 8]. Consequently, laparoscopic appendicectomy has become a well-established technique [9]. In comparison, the current evidence of acute laparoscopic colorectal resection consists of a number of case series and cohort studies, which are limited by their retrospective nature, strict patient selection and small sample size [10, 11]. The evidence base for laparoscopic surgery in the elective colorectal surgery is vast [12, 13]. However, applying this evidence to the acute setting is inappropriate due to the varying levels of sepsis, differing patient physiology and potentially

For peer review only

more advanced disease state. The only way to integrate laparoscopic surgery in the algorithm for acute colorectal pathology is to evaluate its safety and efficacy within the remit of a randomised controlled trial.

Surgical trials have been traditionally deemed to be difficult to undertake due to a range of practical and methodological challenges, including difficulties in recruitment, randomisation and lack of surgical equipoise [14]. These issues are further amplified in the emergency setting and therefore it is important to conduct a feasibility trial to assess key trial processes to ensure successful delivery of a future, definitive trial. This protocol paper outlines the LaCeS feasibility trial (Laparoscopic versus Open Colorectal Surgery in the Acute Setting). The trial aims to assess the feasibility, safety and acceptability of performing a large-scale definitive phase III randomised controlled trial comparing emergency laparoscopic with open surgery for acute colorectal pathology.

Methods

Design

The LaCeS feasibility trial is a prospective, multicentre, single blinded, parallel group, pragmatic randomised controlled feasibility trial. At least sixty-six participants will be randomised on an equal basis to receive either laparoscopic or open surgery across 5 UK centres.

Participants will be blinded to the randomisation allocation until 7 days after surgery, or the day of discharge if earlier. Participants will be followed up at pre-specified time intervals; 3 days, 7 days, 30 days, 3 months and 6 months post-operatively. In addition some patients will also be followed up 12 months post-operatively to assess the feasibility of collecting data out to this time point.

Figure 1: Trial Schema

Primary Outcome

The primary outcome measure is recruitment. The trial aims to recruit at least 66 patients over a 15 month period across 5 UK centres, with a steady state of recruitment of 5 patients per month over the last 12 months of the trial period.

For peer review only - http://bmjopen.bmj.com/site/about/guidelines.xhtml

Secondary Outcomes

Key secondary outcomes include:

- To pilot the recruitment and randomisation processes, and assess their acceptability to clinicians and patients within the emergency setting;
- To assess the safety profile of emergency laparoscopic surgery;
- To explore the potential optimal endpoints, either clinical or patient-reported, that could be used as a primary endpoint in a definitive, phase III trial;
- To explore the practical application and success of blinding in the emergency setting;
- To test the feasibility and refine the strategy for collecting patient-reported quality of-life data and resource use data to inform a future economic evaluation.

Study Population

The study population is those presenting to emergency general surgery services with an acute colorectal pathology requiring urgent resectional surgery.

Setting

The study is being undertaken in 5 NHS hospitals with acute general surgery services able to deliver emergency laparoscopic surgery. These hospitals are a mixture of teaching hospitals and district general hospitals.

Eligibility

Patient Inclusion Criteria

- Aged ≥ 18 years.
- Diagnosis of acute colorectal pathology requiring resectional surgery (for example; acute diverticular disease, inflammatory bowel disease and colorectal cancer) confirmed radiologically and/or endoscopically.
- National Confidential Enquiry into Patient Outcome and Death (NCEPOD) classification of urgent [15]
    - Defined as intervention for acute onset or clinical deterioration of potentially life-threatening conditions, for those conditions that may threaten the survival of limb or organ. Normally

For peer review only

within hours of decision to operate, subdivided into NELA categories of 2a (approx. 2-6 hours) or 2b (approx. 6-18 hours).

- Suitable candidate for surgery as judged by the operating surgeon.
- Suitable for laparoscopic and open surgery in the opinion of the operating surgeon.
- Suitable for laparoscopic and open surgery in the opinion of the anaesthetist.
- Informed written consent obtained.
    - In cases where the patient's judgement is considered temporarily impaired in relation to the condition causing their admission e.g. experiencing significant pain/distress/nausea or acute delirium secondary to sepsis, personal consultee advice would be appropriate.

Patient Exclusion Criteria

- Haemodynamic instability requiring inotropic support
- Acute non-colorectal pathology (for example; adhesional small bowel obstruction, appendicitis, peptic ulcer disease)
- Hand-assisted laparoscopic surgery
- Laparoscopy and peritoneal lavage alone for colorectal pathology
- Insertion of an endoscopic stent followed by laparoscopic resection for colorectal pathology
- Patients undergoing surgery for complications of elective colorectal operations
- Pregnancy
- Pre-existing cognitive impairment
- Currently participating in another surgical trial

Recruitment and Randomisation Process

All patients with suspected acute colorectal pathology will be assessed clinically, radiologically and/or endoscopically as per best clinical practice. Following confirmation of clinical **and** radiological/endoscopic diagnosis of an acute colorectal pathology requiring resection, patients will be approached for participation in the trial. Patients will only be approached for potential participation between the hours of 08:00 – 22:00.

Patients who are deemed to have temporary impairment in their judgement (temporary lack of mental capacity), related to their condition (e.g. experiencing significant pain/distress/nausea or acute delirium secondary to

For peer review only

sepsis), can be entered into the trial if a personal consultee can be identified to advise about trial entry. A personal consultee will ideally be a family member or partner, and will be informed of all key trial processes. Once the patient regains capacity written informed consent will be requested from the patient for on going participation within the trial. Given the emergency nature of the trial, the time available to consider participation will be shorter than in the elective setting. Patients and personal consultees will be given as long as they need to consider participation in the trial, ideally this will be at least 2 hours.

Following appropriate surgical and anaesthetic assessment and confirmation of the clinical diagnosis, patients will be randomised using a telephone or on-line randomisation system, on a 1:1 basis to receive either laparoscopic or open surgery. Patients will be stratified to one of the two arms according to intended consultant surgeon in charge, age, body mass index, ASA status, nature of underlying pathology and intended surgical procedure.

Trial Interventions

Surgery

For the purposes of this pragmatic trial, surgery, either open or laparoscopic, will be undertaken in accordance with local standard practice. Laparoscopic surgery includes the use of multi-port and single-port incisions to establish pneumoperitoneum to enable surgical resection. Conversion to an open operation is defined as the use of a midline laparotomy wound for any part of the colorectal dissection. The use of a limited laparotomy wound to facilitate specimen extraction is permissible.

Blinding

The process of blinding this patient population within the emergency setting will be piloted in this feasibility study. Participants will be blinded to the randomisation allocation for 7 days post-operatively, or until the day of discharge if earlier. Hypoallergenic dressings will be applied to mimic the distribution of the midline laparotomy wound and lateral port site wounds. To assess the success of the blinding protocol the Bang Blinding Index will be used to calculate the proportion of un-blinded participants in the trial on Day 7 post-operatively[16].

Outcome Assessment

For peer review only

Primary Outcome – Recruitment

The primary outcome of this trial is recruitment. Data logs will be kept to assess:

- the number of patients screened for eligibility,
- the proportion of eligible patients consenting to participation and reasons for non-participation,
- the proportion of consenting patients undergoing randomisation and reasons for non-randomisation,
- the proportion of patients not receiving their randomised allocation and the reasons for this.

The combination of quantitative and qualitative data regarding recruitment will enable us to understand the potential pool of eligible patients and reasons for non-participation and withdrawal throughout the recuitment process. This will enable us to further refine and develop our recruitment and randomisation processes for a definitive, phase III trial.

Secondary Outcomes

Safety

To assess the safety profile of acute laparoscopic surgery the following outcomes will be assessed: conversion rates from laparoscopic to open surgery, intra-operative and post-operative complication rates, the severity of post-operative complications using the Clavien-Dindo grading system, the incidence of patient safety indicators and 30 day post-operative mortality rates.

End-point Evaluation to identify the optimal primary endpoint(s) for a definitive phase III trial

A range of key outcomes will be collected, including:

- Clinical outcomes including length of HDU/ICU stay, length of hospital stay, resumption of gastrointestinal function and oral intake, opioid analgesic use, re-operation rates and re-admission rates and details regarding histopathology of the resected specimen.
- Patient reported health-related quality of life data using the Gastrointestinal Quality of Life Index (GIQLI), the SF-12® Health Survey, pain scores using the Brief Pain Inventory (BPI), and the EQ-5D-5L™.
- Resource use using dedicated patient reported and site completed health economics questionnaires to measure primary and secondary healthcare service use.

For peer review only

- Patient and clinician acceptability of trial processes and procedures using in-depth qualitative interviews and a dedicated patient feedback questionnaire.

These candidate endpoints will be explored quantitatively and qualitatively to assess for completion rates, generate data to inform future power calculations and identify which endpoint will be of most meaning and value to clinicians and patients as a primary end point(s) for a definitive phase III trial. Candidate endpoints will be collected at various times during the course of the trial (Table 1). Trial follow up will cease when the last participant reaches 6 months post-randomisation.

Table 1: Schedule of Events

	Pre-trial Diagnostics	Baseline	Operative	3 day Post-op Review	7 day Post-op Review	30 day Post-op Review	3, 6 and 12* months Post-operative Assessment
Radiological/endoscopic diagnosis	✓						
Medical assessment		✓		✓	✓	✓	✓
Participant completed questionnaires		✓		✓	✓	✓	✓
Operative details			✓				
Complications			✓			✓	✓
Patient Safety Indicators					✓ At discharge		✓**
Patient feedback questionnaire					✓		
Blinding Questionnaire					✓		
Resource usage			✓		✓ At discharge		

* Trial follow up will cease when the last participant reaches 6 months post-randomisation

Qualitative Sub-study

Trial processes and their acceptability to clinicians and patients will be assessed using semi-structured, in depth qualitative interviews to optimise and design strategies for a definitive, phase III trial. Clinicians will be interviewed regarding overall trial processes, recruitment in the emergency setting, and potential primary endpoints for a future Phase III trial. Patients will be interviewed to identify any issues with the randomisation process, preferential bias for one type of surgery, reasons for non-participation or withdrawal, refusal of treatment allocation and burden of participation.

For peer review only

Sample Size Calculation and Statistical Analysis

The sample size has been chosen to allow the estimation of the parameters of interest to the necessary degree of precision, following the recommended rule-of-thumb of 30 participants per arm [17]. The sample size has been calculated to account for a 10% attrition rate and aims to recruit at least 66 patients. This sample size will allow the estimation of morbidity and mortality rates with the laparoscopic arm with 95% 2-sided confidence intervals of at most ±17%, allowing its safety profile to be demonstrated. Achievement of this recruitment target will also demonstrate feasibility of a likely required recruitment rate for a successful definitive, phase III trial.

The feasibility of recruitment and randomisation will be evaluated by summarising the screening, eligibility, consent and randomisation processes, including numbers of participants involved during each stage. Descriptive summaries of the participant recruitment pathways at the five recruiting centres will be presented. Reasons for non-participation in the study will be summarised. Participant retention during follow-up, including number of participants completing/withdrawing from the study and reasons for withdrawal, will be presented by treatment arm. Completion rates of data collected at the baseline and follow-up visits will be summarised. The Bang Blinding Index at 7 days and the timings of un-blindings will be reported to inform the feasibility of blinding in a phase III trial. In addition, the relationship between patients, surgical team members and centres will be described to indicate the clustering structure of the feasibility study to inform the design of a phase III trial. The safety profile of each treatment arm will be summarised through descriptive statistics. Mortality rates, intra- and post-operative complication rates, conversion rates and patient safety indicator rates will be reported with 95% confidence intervals. An analysis formally comparing the two treatment arms will not be performed due to the lack of power within this feasibility study, in addition to the purpose of this study.

Ethics

Ethical approval for the trial has been granted by the Health Research Authority, Yorkshire and The Humber, Bradford Leeds Research Ethics Committee. The trial will be performed in accordance with the principles of good clinical practice in clinical trials and the recommendations guiding physicians in biomedical research involving human subjects adopted by the 18th World Medical Assembly, Helsinki, Finland, 1964, amended at the 64th World Medical Association General Assembly, Fortaleza, Brazil, October 2013. Informed written consent will be obtained from the participants (or from personal consultees where appropriate) prior to randomisation into the study. The right of a patient to refuse participation without giving reasons will be

For peer review only

respected. Participants remain free to withdraw at any time from the study without giving reasons and without prejudicing his/her further treatment.

Dissemination

The results of this trial will be presented at relevant colorectal scientific meetings and will be published in peer-reviewed journals.

Discussion

There is a lack of high quality evidence on laparoscopic surgery for emergency colorectal resection. There are a number of well documented challenges in undertaking emergency surgery trials, including issues with recruitment, safety and surgical equipoise [18-20]. The LaCeS feasibility trial is a necessary requirement prior to embarking on a definitive, phase III trial. Conducting this feasibility trial with an embedded qualitative study will enable a greater understanding of trial processes and their acceptability, thus allowing refinement of methodology and infrastructure for a planned, robust, definitive trial.

This feasibility trial is the first of its kind to assess the role of resectional laparoscopic surgery in the acute colorectal setting. The trial aims to assess the role of blinding in the acute clinical scenario, the inclusion of patients with temporary loss of capacity and aims to determine the barriers to recruitment and participation within this framework. The evidence generated from this trial will not only help inform the design of a definitive, phase III trial, but will also help inform future methodological work in recruiting and randomising patients in the emergency setting. Emergency surgery research, and in particular acute colorectal surgery research, has been limited to individual case series and cohort studies, due to perceived difficulties in recruitment, randomisation and retention of patients. The LaCeS feasibility trial will try to understand these issues and offer solutions to help overcome them through consultation with participating surgeons, patients, the trial management group and the trial steering committee. This will lead to the design of a pragmatic, phase III trial, which will reflect the opinions of all key stakeholders.

Contributorship:

DH/KG/HC - protocol writing
HM - statistical input and analysis

For peer review only

DH/CM/DB/BG/PS- clinical input
DB/BG/PS - manuscript review
MT - qualitative input and analysis
JO - health economics evaluation and input
AV - PPI input and manuscript review
JB/PMS - overall review
JB/VH - methodological input

Competing Interests: None

Data Sharing: None available at present

Funding :

"This report is independent research funded by the National Institute for Health Research, Research for Patient Benefit Programme, "Laparoscopic versus Open Colorectal Emergency Surgery: The LACES Feasibility Study", PB-PG-0614-3409. The views expressed in this publication are those of the author(s) and not necessarily those of the NHS, the National Institute for Health Research or the Department of Health."

1. (SEPHO) SEPHO. General surgery: emergency in England 2002/3 to 2004/5. A geographical profile of admissions. 2006.
2. Dawson EJ, Paterson-Brown S. Emergency general surgery and the implications for specialisation. Surgeon 2004; 2: 165-170.
3. Mai-Phan TA, Patel B, Walsh M et al. Emergency room surgical workload in an inner city UK teaching hospital. World J Emerg Surg 2008; 3: 19.
4. al-Ayoubi F, Eriksson H, Myrelid P et al. Distribution of emergency operations and trauma in a Swedish hospital: need for reorganisation of acute surgical care? Scand J Trauma Resusc Emerg Med 2012; 20: 66.
5. Symons NR, Moorthy K, Almoudaris AM et al. Mortality in high-risk emergency general surgical admissions. Br J Surg 2013; 100: 1318-1325.
6. Audit NEL. The Second Patient Report of the National Emergency Laparotomy Audit (NELA). In. 2016.
7. Wei HB, Huang JL, Zheng ZH et al. Laparoscopic versus open appendectomy: a prospective randomized comparison. Surg Endosc 2010; 24: 266-269.
8. McAnena OJ, Austin O, O'Connell PR et al. Laparoscopic versus open appendicectomy: a prospective evaluation. Br J Surg 1992; 79: 818-820.
9. National Surgical Research C. Multicentre observational study of performance variation in provision and outcome of emergency appendicectomy. Br J Surg 2013; 100: 1240-1252.
10. Harji DP, Griffiths B, Burke D, Sagar PM. Systematic review of emergency laparoscopic colorectal resection. Br J Surg 2014; 101: e126-133.
11. Agresta F, Ansaloni L, Baiocchi GL et al. Laparoscopic approach to acute abdomen from the Consensus Development Conference of the Societa Italiana di Chirurgia Endoscopica e nuove tecnologie (SICE), Associazione Chirurghi Ospedalieri Italiani (ACOI), Societa Italiana di Chirurgia (SIC), Societa Italiana di Chirurgia d'Urgenza e del Trauma (SICUT), Societa Italiana di Chirurgia nell'Ospedalita Privata (SICOP), and the European Association for Endoscopic Surgery (EAES). Surg Endosc 2012; 26: 2134-2164.

For peer review only

12. Guillou PJ, Quirke P, Thorpe H et al. Short-term endpoints of conventional versus laparoscopic-assisted surgery in patients with colorectal cancer (MRC CLASICC trial): multicentre, randomised controlled trial. Lancet 2005; 365: 1718-1726.

13. Hazebroek EJ, Color Study G. COLOR: a randomized clinical trial comparing laparoscopic and open resection for colon cancer. Surg Endosc 2002; 16: 949-953.

14. Kaur G, Hutchison I, Mehanna H et al. Barriers to recruitment for surgical trials in head and neck oncology: a survey of trial investigators. BMJ Open 2013; 3.

15. Intervention. NCo. 2004.

16. Bang H, Ni L, Davis CE. Assessment of blinding in clinical trials. Control Clin Trials 2004; 25: 143-156.

17. Lancaster GA, Dodd S, Williamson PR. Design and analysis of pilot studies: recommendations for good practice. J Eval Clin Pract 2004; 10: 307-312.

18. Binda GA, Karas JR, Serventi A et al. Primary anastomosis vs nonrestorative resection for perforated diverticulitis with peritonitis: a prematurely terminated randomized controlled trial. Colorectal Dis 2012; 14: 1403-1410.

19. Oberkofler CE, Rickenbacher A, Raptis DA et al. A multicenter randomized clinical trial of primary anastomosis or Hartmann's procedure for perforated left colonic diverticulitis with purulent or fecal peritonitis. Ann Surg 2012; 256: 819-826; discussion 826-817.

20. Vennix S, Musters GD, Mulder IM et al. Laparoscopic peritoneal lavage or sigmoidectomy for perforated diverticulitis with purulent peritonitis: a multicentre, parallel-group, randomised, open-label trial. Lancet 2015; 386: 1269-1277.

For peer review only

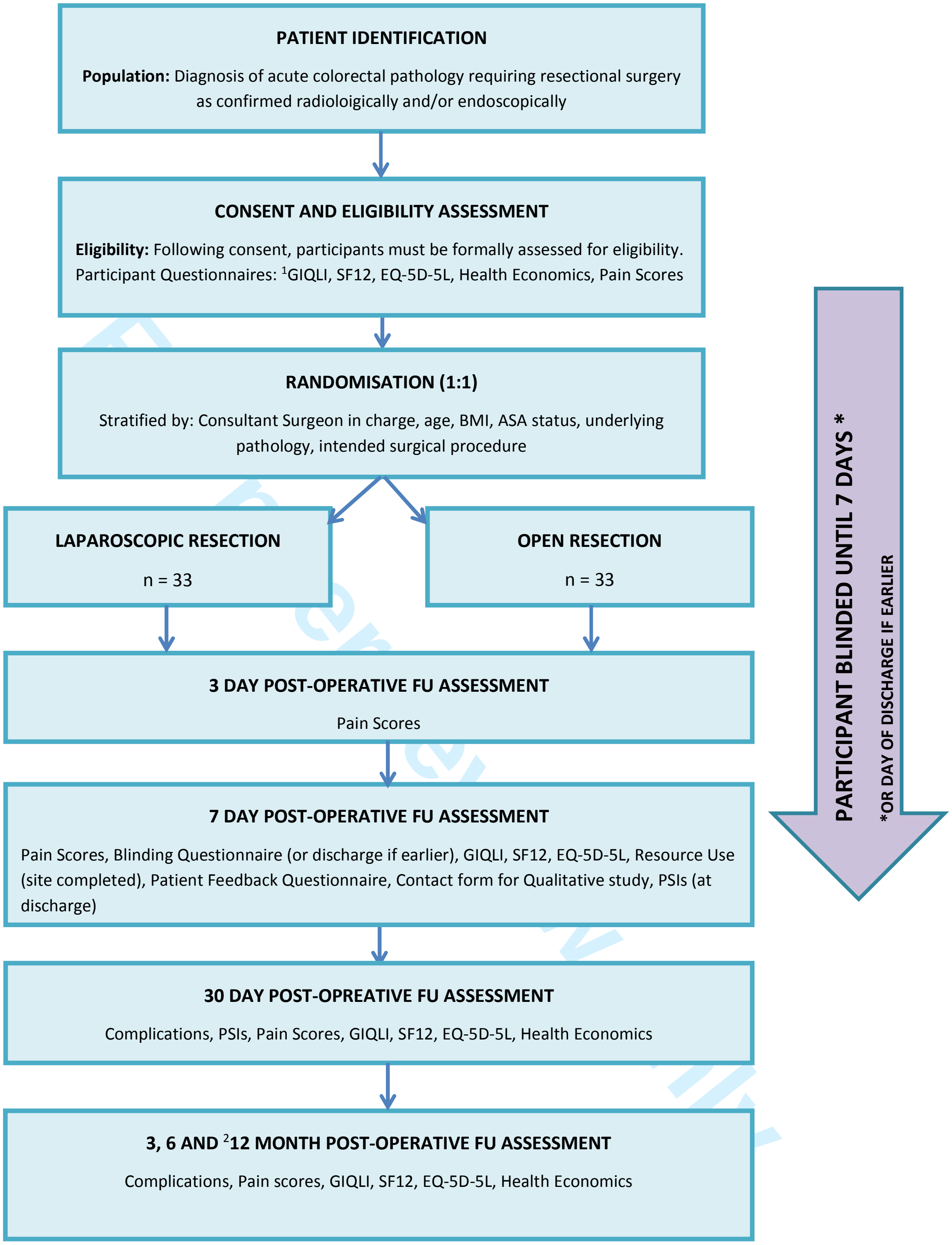

[1] Gastrointestinal Quality of Life Index
[2] Trial follow up will cease when the last randomised patient reaches 6 months post randomisation therefore not all patients will reach 12 months follow up

SPIRIT 2013 Checklist: Recommended items to address in a clinical trial protocol and related documents*

Section/item	Page	Item No	Description
**Administrative information**			
Title	x	1	Descriptive title identifying the study design, population, interventions, and, if applicable, trial acronym
Trial registration	x	2a	Trial identifier and registry name. If not yet registered, name of intended registry
		2b	All items from the World Health Organization Trial Registration Data Set
Protocol version		3	Date and version identifier
Funding	X	4	Sources and types of financial, material, and other support
Roles and responsibilities	1-2	5a	Names, affiliations, and roles of protocol contributors
	x	5b	Name and contact information for the trial sponsor
		5c	Role of study sponsor and funders, if any, in study design; collection, management, analysis, and interpretation of data; writing of the report; and the decision to submit the report for publication, including whether they will have ultimate authority over any of these activities
		5d	Composition, roles, and responsibilities of the coordinating centre, steering committee, endpoint adjudication committee, data management team, and other individuals or groups overseeing the trial, if applicable (see Item 21a for data monitoring committee)
**Introduction**			
Background and rationale	4	6a	Description of research question and justification for undertaking the trial, including summary of relevant studies (published and unpublished) examining benefits and harms for each intervention
	4-5	6b	Explanation for choice of comparators
Objectives	5	7	Specific objectives or hypotheses
Trial design	5	8	Description of trial design including type of trial (eg, parallel group, crossover, factorial, single group), allocation ratio, and framework (eg, superiority, equivalence, noninferiority, exploratory)

For peer review only

Methods: Participants, interventions, and outcomes

Study setting	6	9	Description of study settings (eg, community clinic, academic hospital) and list of countries where data will be collected. Reference to where list of study sites can be obtained
Eligibility criteria	6	10	Inclusion and exclusion criteria for participants. If applicable, eligibility criteria for study centres and individuals who will perform the interventions (eg, surgeons, psychotherapists)
Interventions	8	11a	Interventions for each group with sufficient detail to allow replication, including how and when they will be administered
		11b	Criteria for discontinuing or modifying allocated interventions for a given trial participant (eg, drug dose change in response to harms, participant request, or improving/worsening disease)
		11c	Strategies to improve adherence to intervention protocols, and any procedures for monitoring adherence (eg, drug tablet return, laboratory tests)
		11d	Relevant concomitant care and interventions that are permitted or prohibited during the trial
Outcomes	9 - 10	12	Primary, secondary, and other outcomes, including the specific measurement variable (eg, systolic blood pressure), analysis metric (eg, change from baseline, final value, time to event), method of aggregation (eg, median, proportion), and time point for each outcome. Explanation of the clinical relevance of chosen efficacy and harm outcomes is strongly recommended
Participant timeline	10	13	Time schedule of enrolment, interventions (including any run-ins and washouts), assessments, and visits for participants. A schematic diagram is highly recommended (see Figure)
Sample size	11	14	Estimated number of participants needed to achieve study objectives and how it was determined, including clinical and statistical assumptions supporting any sample size calculations
Recruitment	7 and 10	15	Strategies for achieving adequate participant enrolment to reach target sample size

Methods: Assignment of interventions (for controlled trials)

Allocation:			
Sequence generation	7	16a	Method of generating the allocation sequence (eg, computer-generated random numbers), and list of any factors for stratification. To reduce predictability of a random sequence, details of any planned restriction (eg, blocking) should be provided in a separate document that is unavailable to those who enrol participants or assign interventions

Allocation concealment mechanism		16b	Mechanism of implementing the allocation sequence (eg, central telephone; sequentially numbered, opaque, sealed envelopes), describing any steps to conceal the sequence until interventions are assigned
Implementation		16c	Who will generate the allocation sequence, who will enrol participants, and who will assign participants to interventions
Blinding (masking)	8	17a	Who will be blinded after assignment to interventions (eg, trial participants, care providers, outcome assessors, data analysts), and how
		17b	If blinded, circumstances under which unblinding is permissible, and procedure for revealing a participant's allocated intervention during the trial
**Methods: Data collection, management, and analysis**			
Data collection methods	9 and 10	18a	Plans for assessment and collection of outcome, baseline, and other trial data, including any related processes to promote data quality (eg, duplicate measurements, training of assessors) and a description of study instruments (eg, questionnaires, laboratory tests) along with their reliability and validity, if known. Reference to where data collection forms can be found, if not in the protocol
	12	18b	Plans to promote participant retention and complete follow-up, including list of any outcome data to be collected for participants who discontinue or deviate from intervention protocols
Data management		19	Plans for data entry, coding, security, and storage, including any related processes to promote data quality (eg, double data entry; range checks for data values). Reference to where details of data management procedures can be found, if not in the protocol
Statistical methods	11	20a	Statistical methods for analysing primary and secondary outcomes. Reference to where other details of the statistical analysis plan can be found, if not in the protocol
		20b	Methods for any additional analyses (eg, subgroup and adjusted analyses)
		20c	Definition of analysis population relating to protocol non-adherence (eg, as randomised analysis), and any statistical methods to handle missing data (eg, multiple imputation)
**Methods: Monitoring**			
Data monitoring		21a	Composition of data monitoring committee (DMC); summary of its role and reporting structure; statement of whether it is independent from the sponsor and competing interests; and reference to where further details about its charter can be found, if not in the protocol. Alternatively, an explanation of why a DMC is not needed

		21b	Description of any interim analyses and stopping guidelines, including who will have access to these interim results and make the final decision to terminate the trial
Harms		22	Plans for collecting, assessing, reporting, and managing solicited and spontaneously reported adverse events and other unintended effects of trial interventions or trial conduct
Auditing		23	Frequency and procedures for auditing trial conduct, if any, and whether the process will be independent from investigators and the sponsor
**Ethics and dissemination**			
Research ethics approval	11	24	Plans for seeking research ethics committee/institutional review board (REC/IRB) approval
Protocol amendments		25	Plans for communicating important protocol modifications (eg, changes to eligibility criteria, outcomes, analyses) to relevant parties (eg, investigators, REC/IRBs, trial participants, trial registries, journals, regulators)
Consent or assent	7/8	26a	Who will obtain informed consent or assent from potential trial participants or authorised surrogates, and how (see Item 32)
		26b	Additional consent provisions for collection and use of participant data and biological specimens in ancillary studies, if applicable
Confidentiality		27	How personal information about potential and enrolled participants will be collected, shared, and maintained in order to protect confidentiality before, during, and after the trial
Declaration of interests	12	28	Financial and other competing interests for principal investigators for the overall trial and each study site
Access to data		29	Statement of who will have access to the final trial dataset, and disclosure of contractual agreements that limit such access for investigators
Ancillary and post-trial care		30	Provisions, if any, for ancillary and post-trial care, and for compensation to those who suffer harm from trial participation
Dissemination policy	12	31a	Plans for investigators and sponsor to communicate trial results to participants, healthcare professionals, the public, and other relevant groups (eg, via publication, reporting in results databases, or other data sharing arrangements), including any publication restrictions
		31b	Authorship eligibility guidelines and any intended use of professional writers
		31c	Plans, if any, for granting public access to the full protocol, participant-level dataset, and statistical code

For peer review only

Appendices

Informed consent materials	32	Model consent form and other related documentation given to participants and authorised surrogates
Biological specimens	33	Plans for collection, laboratory evaluation, and storage of biological specimens for genetic or molecular analysis in the current trial and for future use in ancillary studies, if applicable

*It is strongly recommended that this checklist be read in conjunction with the SPIRIT 2013 Explanation & Elaboration for important clarification on the items. Amendments to the protocol should be tracked and dated. The SPIRIT checklist is copyrighted by the SPIRIT Group under the Creative Commons "Attribution-NonCommercial-NoDerivs 3.0 Unported" license.

For peer review only

BMJ Open

The feasibility of a multi-centre, randomised controlled trial of laparoscopic versus open colorectal surgery in the acute setting – The LaCeS Feasibility Trial Protocol

Journal:	*BMJ Open*
Manuscript ID	bmjopen-2017-018618.R1
Article Type:	Protocol
Date Submitted by the Author:	10-Oct-2017
Complete List of Authors:	Harji, Deena; Northern Deanery; University of Leeds Clinical Trials Research Unit<br>Marshall, Helen; University of Leeds Clinical Trials Research Unit<br>Gordon, Kathryn; University of Leeds Clinical Trials Research Unit, CTRU<br>Crow, Hannah; University of Leeds Clinical Trials Research Unit<br>Hiley, Victoria; University of Leeds Clinical Trials Research Unit<br>Burke, Dermot; St James' University Hospital, John Goligher Colorectal Department<br>Griffiths, Ben ; Newcastle Upon Tyne Hospitals NHS Foundation Trust<br>Moriarty, Catherine ; St James' University Hospital , John Goligher Colorectal Department<br>Twiddy, Maureen; University of Leeds, Leeds Institute of Health Sciences<br>O'Dwyer, John; University of Leeds Leeds Institute of Health Sciences, Academic Unit of Health Economics<br>Verjee, Azmina<br>Brown, Julia; University of Leeds, Leeds Institute of Clinical Trials Research<br>Sagar , Peter; St James' University Hospital, John Goligher Colorectal Department
**Primary Subject Heading**:	Surgery
Secondary Subject Heading:	Evidence based practice
Keywords:	Colorectal surgery < SURGERY, Clinical trials < THERAPEUTICS, Adult surgery < SURGERY

SCHOLARONE™
Manuscripts

The feasibility of a multi-centre, randomised controlled trial of laparoscopic versus open colorectal surgery in the acute setting – The LaCeS Feasibility Trial Protocol

Corresponding Author

Miss Deena Harji

Northern Deanery, Waterfront 4, Goldcrest Way, Newburn Riverside, Newcastle Upon Tyne, NE15 8NY

Clinical Trials Research Unit, University of Leeds, Leeds

deena_harji@hotmail.com

Co-Authors

Mrs Helen Marshall

Clinical Trials Research Unit, University of Leeds, Leeds

H.C.Marshall@leeds.ac.uk

Ms Katie Gordon,

Clinical Trials Research Unit, University of Leeds, Leeds

k.a.gordon@leeds.ac.uk

Miss Hannah Crow

Clinical Trials Research Unit, University of Leeds, Leeds

h.e.crow@leeds.ac.uk

Mrs Victoria Hiley

Clinical Trials Research Unit, University of Leeds, Leeds

V.Hiley@leeds.ac.uk

Mr Dermot Burke

St James's University Hospital, Beckett Street, Leeds

D.Burke@leeds.ac.uk

Mr Ben Griffiths

For peer review only

Royal Victoria Infirmary, Queen Victoria Road, Newcastle

ben.griffiths@nuth.nhs.uk

Mrs Catherine Moriarty

St James's University Hospital, Beckett Street, Leeds

catherine.moriarty@nhs.net

Dr Maureen Twiddy

Leeds Institute of Health Sciences, University of Leeds, Leeds

M.Twiddy@leeds.ac.uk

Mr John L O'Dwyer

Academic Unit of Health Economics, Leeds Institute of Health Sciences, University of Leeds, Leeds

j.odwyer@leeds.ac.uk

Miss Azmina Verjee

Patient and Public Involvement Representative

azminaverjee@hotmail.com

Professor Julia Brown

Clinical Trials Research Unit, University of Leeds, Leeds

J.M.B.Brown@leeds.ac.uk

Professor Peter Sagar

St James's University Hospital, Beckett Street, Leeds

petersagar@aol.com

Word Count: 2622

Abstract

Introduction

For peer review only

Acute colorectal surgery forms a significant proportion of emergency admissions within the NHS. There is limited evidence to suggest minimally invasive surgery may be associated with improved clinical outcomes in this cohort of patients. Consequently, there is a need to assess the clinical effectiveness and cost-effectiveness of laparoscopic surgery in the acute colorectal setting. However, emergency colorectal surgical trials have previously been difficult to conduct due to issues surrounding recruitment and equipoise. The LaCeS (randomised controlled trial of **La**paroscopic versus open **C**olorectal **S**urgery in the acute setting) feasibility trial will determine the feasibility of conducting a definitive, phase III trial of laparoscopic versus open acute colorectal resection.

Methods and Analysis

The LaCeS feasibility trial is a prospective, multicentre, single blinded, parallel group, pragmatic randomised controlled feasibility trial. Patients will be randomised on a 1:1 basis to receive either laparoscopic or open surgery. The trial aims to recruit at least sixty-six patients from five acute general surgical units across the United Kingdom. Patients over the age of 18 with a diagnosis of acute colorectal pathology requiring resection on clinical and radiological/endoscopic investigations, with an NCEPOD classification of urgent will be considered eligible for participation. The primary outcome is recruitment. Secondary outcomes include assessing the safety profile of laparoscopic surgery using intra- and post-operative complication rates, conversion rates and patient safety indicators as surrogate markers. Clinical, patient-reported costs and outcomes will also be reported. The trial will contain an embedded qualitative study to assess clinician and patient acceptability of trial processes.

Ethics and Dissemination

The LaCeS feasibility trial is approved by the Yorkshire and The Humber, Bradford Leeds Research Ethics Committee (REC reference: 15/YH/0542). The results from the trial will be presented at national and international colorectal conferences and will be submitted for publication to peer-reviewed journals.

Strengths and Limitations

This trial will assess the feasibility and acceptability of conducting a definitive, phase III randomised controlled trial of laparoscopic versus open emergency colorectal resection.

For peer review only

The main challenges regarding recruitment, randomisation, equipoise, blinding and follow-up will be identified through the use of an embedded qualitative study.

The main limitations of this trial are the lack of power to examine efficacy.

Trial Registration

Trial registration number: ISRCTN15681041. Registered on 18th April 2016.

Keywords

Colorectal surgery – feasibility study – randomised controlled trial – emergency surgery – laparoscopic surgery

Background

Emergency general surgery is a huge clinical service, with approximately 1000 Finished Consultant Episodes per 100,000 population/year [1, 2]. Approximately 30% of emergency admissions are secondary to colorectal pathology, namely, colorectal malignancy, inflammatory bowel disease and diverticular disease [3, 4]. More than 30,000 patients undergo an emergency laparotomy for a variety of intra-abdominal pathologies each year within the NHS in England and Wales [5]. In the UK, the National Emergency Laparotomy Audit (NELA) reported outcomes on 23,198 patients, of which 37% underwent an emergency colorectal resection between December 2014 and November 2015 [6]. The burden of emergency surgery is significant, with reports of 30 day postoperative morbidity rates of 33-71% and mortality rates of 14-17%.

The NELA audit reports that the majority of emergency surgery is undertaken using an open approach, with approximately 14% of all emergency abdominal operations commenced laparoscopically, of which only half are completed laparoscopically [6]. The role of laparoscopic surgery in certain acute intra-abdominal pathologies i.e. acute appendicitis has been well elucidated in a number of randomised controlled trials, with reports of improved pain, shorter recovery and reduced length of hospital stay [7, 8]. Consequently, laparoscopic appendicectomy has become a well-established technique [9]. In comparison, the current evidence of acute laparoscopic colorectal resection consists of a number of case series and cohort studies, which are limited by their retrospective nature, strict patient selection and small sample size [10, 11]. The evidence base for laparoscopic surgery in the elective colorectal surgery is vast [12, 13]. However, applying this evidence to the acute setting is inappropriate due to the varying levels of sepsis, differing patient physiology and potentially

For peer review only

more advanced disease state. The only way to integrate laparoscopic surgery in the algorithm for acute colorectal pathology is to evaluate its safety and efficacy within the remit of a randomised controlled trial.

Surgical trials have been traditionally deemed to be difficult to undertake due to a range of practical and methodological challenges, including difficulties in recruitment, randomisation and lack of surgical equipoise [14]. These issues are further amplified in the emergency setting and therefore it is important to conduct a feasibility trial to assess key trial processes to ensure successful delivery of a future, definitive trial. This protocol paper outlines the LaCeS feasibility trial (Laparoscopic versus Open Colorectal Surgery in the Acute Setting). The trial aims to assess the feasibility, safety and acceptability of performing a large-scale definitive phase III randomised controlled trial comparing emergency laparoscopic with open surgery for acute colorectal pathology.

Methods

Design

The LaCeS feasibility trial is a prospective, multicentre, single blinded, parallel group, pragmatic randomised controlled feasibility trial. At least sixty-six participants will be randomised on an equal basis to receive either laparoscopic or open surgery across 5 UK centres.

Participants will be blinded to the randomisation allocation until 7 days after surgery, or the day of discharge if earlier. Participants will be followed up at pre-specified time intervals; 3 days, 7 days, 30 days, 3 months and 6 months post-operatively. In addition some patients will also be followed up 12 months post-operatively to assess the feasibility of collecting data out to this time point. The trial schema is outlined in Figure 1.

Primary Outcome

The primary outcome measure is recruitment. The trial aims to recruit at least 66 patients over a 15 month period across 5 UK centres, with a steady state of recruitment of 5 patients per month over the last 12 months of the trial period.

Secondary Outcomes

For peer review only

Key secondary outcomes include:

- To pilot the recruitment and randomisation processes, and assess their acceptability to clinicians and patients within the emergency setting;
- To assess the safety profile of emergency laparoscopic surgery;
- To explore the potential optimal endpoints, either clinical or patient-reported, that could be used as a primary endpoint in a definitive, phase III trial;
- To explore the practical application and success of blinding in the emergency setting;
- To test the feasibility and refine the strategy for collecting patient-reported quality of-life data and resource use data to inform a future economic evaluation.

Study Population

The study population is those presenting to emergency general surgery services with an acute colorectal pathology requiring urgent resectional surgery.

Setting

The study is being undertaken in 5 NHS hospitals with acute general surgery services able to deliver emergency laparoscopic surgery. These hospitals are a mixture of teaching hospitals and district general hospitals with dedicated emergency surgery radiology services.

Eligibility

Patient Inclusion Criteria

- Aged ≥ 18 years.
- Diagnosis of acute colorectal pathology requiring resectional surgery (for example; acute diverticular disease, inflammatory bowel disease and colorectal cancer) confirmed radiologically and/or endoscopically.
- National Confidential Enquiry into Patient Outcome and Death (NCEPOD) classification of urgent [15]
    - Defined as intervention for acute onset or clinical deterioration of potentially life-threatening conditions, for those conditions that may threaten the survival of limb or organ. Normally within hours of decision to operate, subdivided into NELA categories of 2a (approx. 2-6 hours) or 2b (approx. 6-18 hours).

For peer review only

- Suitable candidate for surgery as judged by the operating surgeon.
- Suitable for laparoscopic and open surgery in the opinion of the operating surgeon.
- Suitable for laparoscopic and open surgery in the opinion of the anaesthetist.
- Informed written consent obtained.
    - In cases where the patient's judgement is considered temporarily impaired in relation to the condition causing their admission e.g. experiencing significant pain/distress/nausea or acute delirium secondary to sepsis, personal consultee advice would be appropriate.

Patient Exclusion Criteria

- Haemodynamic instability requiring inotropic support
- Acute non-colorectal pathology (for example; adhesional small bowel obstruction, appendicitis, peptic ulcer disease)
- Hand-assisted laparoscopic surgery
- Laparoscopy and peritoneal lavage alone for colorectal pathology
- Insertion of an endoscopic stent followed by laparoscopic resection for colorectal pathology
- Patients undergoing surgery for complications of elective colorectal operations
- Pregnancy
- Pre-existing cognitive impairment
- Currently participating in another surgical trial

Site Eligibility

The trial will be performed as a multicentre collaboration within the UK across approximately 5 sites. Participation of sites will be dependent upon the following criteria:

- Has dedicated emergency surgery services with appropriate provisions for emergency laparoscopic surgery
- Has dedicated elective laparoscopic colorectal surgery services
- Established previous involvement in clinical trials
- Anticipating to recruit at least 2-3 patients per month

Surgeon Eligibility

All participating consultant surgeons must have a subspecialist interest in colorectal surgery and have performed a minimum of 50 laparoscopic colorectal resections and must perform at least 20 laparoscopic resections a year, with equivalent experience with open surgery (this can include procedures in both the emergency and elective setting).

For peer review only

Recruitment and Randomisation Process

All patients with suspected acute colorectal pathology will be assessed clinically, radiologically and/or endoscopically as per best clinical practice. Following confirmation of clinical **and** radiological/endoscopic diagnosis of an acute colorectal pathology requiring resection, patients will be approached for participation in the trial. Patients will only be approached for potential participation between the hours of 08:00 – 22:00.

Patients who are deemed to have temporary impairment in their judgement (temporary lack of mental capacity), related to their condition (e.g. experiencing significant pain/distress/nausea or acute delirium secondary to sepsis), can be entered into the trial if a personal consultee can be identified to advise about trial entry. A personal consultee will ideally be a family member or partner, and will be informed of all key trial processes. Once the patient regains capacity written informed consent will be requested from the patient for on going participation within the trial. Given the emergency nature of the trial, the time available to consider participation will be shorter than in the elective setting. Patients and personal consultees will be given as long as they need to consider participation in the trial, ideally this will be at least 2 hours.

Following appropriate surgical and anaesthetic assessment and confirmation of the clinical diagnosis, patients will be randomised using a telephone or on-line randomisation system, on a 1:1 basis to receive either laparoscopic or open surgery. Patients will be stratified to one of the two arms according to intended consultant surgeon in charge, age, body mass index, ASA status, nature of underlying pathology and intended surgical procedure.

Trial Interventions

Surgery

For the purposes of this pragmatic trial, surgery, either open or laparoscopic, will be undertaken in accordance with local standard practice. Laparoscopic surgery includes the use of multi-port and single-port incisions to establish pneumoperitoneum to enable surgical resection. Conversion to an open operation is defined as the use of a midline laparotomy wound for any part of the colorectal dissection. The use of a limited laparotomy wound to facilitate specimen extraction is permissible.

For peer review only

Blinding

The process of blinding this patient population within the emergency setting will be piloted in this feasibility study. Participants will be blinded to the randomisation allocation for 7 days post-operatively, or until the day of discharge if earlier. Hypoallergenic dressings will be applied to mimic the distribution of the midline laparotomy wound and lateral port site wounds. To assess the success of the blinding protocol the Bang Blinding Index will be used to calculate the proportion of un-blinded participants in the trial on Day 7 post-operatively[16].

Outcome Assessment

Primary Outcome – Recruitment

The primary outcome of this trial is recruitment. Data logs will be kept to assess:

- the number of patients screened for eligibility,
- the proportion of eligible patients consenting to participation and reasons for non-participation,
- the proportion of consenting patients undergoing randomisation and reasons for non-randomisation,
- the proportion of patients not receiving their randomised allocation and the reasons for this.

The combination of quanitative and qualitative data regarding recruitment will enable us to understand the potential pool of eligible patients and reasons for non-participation and withdrawal throughout the recuitment process. This will enable us to further refine and develop our recruitment and randomisation processes for a definitive, phase III trial.

Secondary Outcomes

Safety

To assess the safety profile of acute laparoscopic surgery the following outcomes will be assessed: conversion rates from laparoscopic to open surgery, intra-operative and post-operative complication rates, the severity of post-operative complications using the Clavien-Dindo grading system, the incidence of patient safety indicators and 30 day post-operative mortality rates.

End-point Evaluation to identify the optimal primary endpoint(s) for a definitive phase III trial

For peer review only

A range of key outcomes will be collected, including:

- Clinical outcomes including length of HDU/ICU stay, length of hospital stay, resumption of gastrointestinal function and oral intake, opioid analgesic use, re-operation rates and re-admission rates and details regarding histopathology of the resected specimen.
- Patient reported health-related quality of life data using the Gastrointestinal Quality of Life Index (GIQLI), the SF-12® Health Survey, pain scores using the Brief Pain Inventory (BPI), and the EQ-5D-5L™.
- Resource use using dedicated patient reported and site completed health economics questionnaires to measure primary and secondary healthcare service use.
- Patient and clinician acceptability of trial processes and procedures using in-depth qualitative interviews and a dedicated patient feedback questionnaire.

These candidate endpoints will be explored quantitatively and qualitatively to assess for completion rates, generate data to inform future power calculations and identify which endpoint will be of most meaning and value to clinicians and patients as a primary end point(s) for a definitive phase III trial. Candidate endpoints will be collected at various times during the course of the trial (Table 1). Trial follow up will cease when the last participant reaches 6 months post-randomisation.

Table 1: Schedule of Events

	Pre-trial Diagnostics	Baseline	Operative	3 day Post-op Review	7 day Post-op Review	30 day Post-op Review	3, 6 and 12* months Post-operative Assessment
Radiological/endoscopic diagnosis	✓						
Medical assessment		✓		✓	✓	✓	✓
Participant completed questionnaires		✓		✓	✓	✓	✓
Operative details			✓				
Complications			✓			✓	✓
Patient Safety Indicators					✓ At discharge		✓**
Patient feedback questionnaire					✓		
Blinding Questionnaire					✓		
Resource usage			✓		✓ At discharge		

* Trial follow up will cease when the last participant reaches 6 months post-randomisation

For peer review only

Qualitative Sub-study

Trial processes and their acceptability to clinicians and patients will be assessed using semi-structured, in depth qualitative interviews to optimise and design strategies for a definitive, phase III trial. Clinicians will be interviewed regarding overall trial processes, recruitment in the emergency setting, and potential primary endpoints for a future Phase III trial. Patients will be interviewed to identify any issues with the randomisation process, preferential bias for one type of surgery, reasons for non-participation or withdrawal, refusal of treatment allocation and burden of participation.

Sample Size Calculation and Statistical Analysis

The sample size has been chosen to allow the estimation of the parameters of interest to the necessary degree of precision, following the recommended rule-of-thumb of 30 participants per arm [17]. The sample size has been calculated to account for a 10% attrition rate and aims to recruit at least 66 patients. This sample size will allow the estimation of morbidity and mortality rates with the laparoscopic arm with 95% 2-sided confidence intervals of at most ±17%, allowing its safety profile to be demonstrated. Achievement of this recruitment target will also demonstrate feasibility of a likely required recruitment rate for a successful definitive, phase III trial.

The feasibility of recruitment and randomisation will be evaluated by summarising the screening, eligibility, consent and randomisation processes, including numbers of participants involved during each stage. Descriptive summaries of the participant recruitment pathways at the five recruiting centres will be presented. Reasons for non-participation in the study will be summarised. Participant retention during follow-up, including number of participants completing/withdrawing from the study and reasons for withdrawal, will be presented by treatment arm. Completion rates of data collected at the baseline and follow-up visits will be summarised. The Bang Blinding Index at 7 days and the timings of un-blindings will be reported to inform the feasibility of blinding in a phase III trial. In addition, the relationship between patients, surgical team members and centres will be described to indicate the clustering structure of the feasibility study to inform the design of a phase III trial. The safety profile of each treatment arm will be summarised through descriptive statistics. Mortality rates, intra- and post-operative complication rates, conversion rates and patient safety indicator rates will be reported with 95% confidence intervals. All analyses will be conducted on an intention to treat basis. An analysis formally

For peer review only

comparing the two treatment arms will not be performed due to the lack of power within this feasibility study, in addition to the purpose of this study.

Ethics

Ethical approval for the trial has been granted by the Health Research Authority, Yorkshire and The Humber, Bradford Leeds Research Ethics Committee. The trial will be performed in accordance with the principles of good clinical practice in clinical trials and the recommendations guiding physicians in biomedical research involving human subjects adopted by the 18th World Medical Assembly, Helsinki, Finland, 1964, amended at the 64th World Medical Association General Assembly, Fortaleza, Brazil, October 2013. Informed written consent will be obtained from the participants (or from personal consultees where appropriate) prior to randomisation into the study. The right of a patient to refuse participation without giving reasons will be respected. Participants remain free to withdraw at any time from the study without giving reasons and without prejudicing his/her further treatment.

Dissemination

The results of this trial will be presented at relevant colorectal scientific meetings and will be published in peer-reviewed journals.

Discussion

There is a lack of high quality evidence on laparoscopic surgery for emergency colorectal resection. There are a number of well documented challenges in undertaking emergency surgery trials, including issues with recruitment, safety and surgical equipoise [18-20]. The LaCeS feasibility trial is a necessary requirement prior to embarking on a definitive, phase III trial. Conducting this feasibility trial with an embedded qualitative study will enable a greater understanding of trial processes and their acceptability, thus allowing refinement of methodology and infrastructure for a planned, robust, definitive trial.

This feasibility trial is the first of its kind to assess the role of resectional laparoscopic surgery in the acute colorectal setting. The trial aims to assess the role of blinding in the acute clinical scenario, the inclusion of patients with temporary loss of capacity and aims to determine the barriers to recruitment and participation within this framework. The evidence generated from this trial will not only help inform the design of a

For peer review only

definitive, phase III trial, but will also help inform future methodological work in recruiting and randomising patients in the emergency setting. Emergency surgery research, and in particular acute colorectal surgery research, has been limited to individual case series and cohort studies, due to perceived difficulties in recruitment, randomisation and retention of patients. The LaCeS feasibility trial will try to understand these issues and offer solutions to help overcome them through consultation with participating surgeons, patients, the trial management group and the trial steering committee. This will lead to the design of a pragmatic, phase III trial, which will reflect the opinions of all key stakeholders.

Figure 1: Trial Schema

Contributorship:

DH/KG/HC - protocol writing
HM - statistical input and analysis
DH/CM/DB/BG/PS- clinical input
DB/BG/PS - manuscript review
MT - qualitative input and analysis
JO - health economics evaluation and input
AV - PPI input and manuscript review
JB/PMS - overall review
JB/VH - methodological input

Competing Interests: None

Data Sharing: None available at present

Funding :

"This report is independent research funded by the National Institute for Health Research, Research for Patient Benefit Programme, "Laparoscopic versus Open Colorectal Emergency Surgery: The LACES Feasibility Study", PB-PG-0614-3409. The views expressed in this publication are those of the author(s) and not necessarily those of the NHS, the National Institute for Health Research or the Department of Health."

1. (SEPHO) SEPHO. General surgery: emergency in England 2002/3 to 2004/5. A geographical profile of admissions. 2006.
2. Dawson EJ, Paterson-Brown S. Emergency general surgery and the implications for specialisation. Surgeon 2004; 2: 165-170.
3. Mai-Phan TA, Patel B, Walsh M et al. Emergency room surgical workload in an inner city UK teaching hospital. World J Emerg Surg 2008; 3: 19.

For peer review only

4. al-Ayoubi F, Eriksson H, Myrelid P et al. Distribution of emergency operations and trauma in a Swedish hospital: need for reorganisation of acute surgical care? Scand J Trauma Resusc Emerg Med 2012; 20: 66.
5. Symons NR, Moorthy K, Almoudaris AM et al. Mortality in high-risk emergency general surgical admissions. Br J Surg 2013; 100: 1318-1325.
6. Audit NEL. The Second Patient Report of the National Emergency Laparotomy Audit (NELA). In. 2016.
7. Wei HB, Huang JL, Zheng ZH et al. Laparoscopic versus open appendectomy: a prospective randomized comparison. Surg Endosc 2010; 24: 266-269.
8. McAnena OJ, Austin O, O'Connell PR et al. Laparoscopic versus open appendicectomy: a prospective evaluation. Br J Surg 1992; 79: 818-820.
9. National Surgical Research C. Multicentre observational study of performance variation in provision and outcome of emergency appendicectomy. Br J Surg 2013; 100: 1240-1252.
10. Harji DP, Griffiths B, Burke D, Sagar PM. Systematic review of emergency laparoscopic colorectal resection. Br J Surg 2014; 101: e126-133.
11. Agresta F, Ansaloni L, Baiocchi GL et al. Laparoscopic approach to acute abdomen from the Consensus Development Conference of the Societa Italiana di Chirurgia Endoscopica e nuove tecnologie (SICE), Associazione Chirurghi Ospedalieri Italiani (ACOI), Societa Italiana di Chirurgia (SIC), Societa Italiana di Chirurgia d'Urgenza e del Trauma (SICUT), Societa Italiana di Chirurgia nell'Ospedalita Privata (SICOP), and the European Association for Endoscopic Surgery (EAES). Surg Endosc 2012; 26: 2134-2164.
12. Guillou PJ, Quirke P, Thorpe H et al. Short-term endpoints of conventional versus laparoscopic-assisted surgery in patients with colorectal cancer (MRC CLASICC trial): multicentre, randomised controlled trial. Lancet 2005; 365: 1718-1726.
13. Hazebroek EJ, Color Study G. COLOR: a randomized clinical trial comparing laparoscopic and open resection for colon cancer. Surg Endosc 2002; 16: 949-953.
14. Kaur G, Hutchison I, Mehanna H et al. Barriers to recruitment for surgical trials in head and neck oncology: a survey of trial investigators. BMJ Open 2013; 3.
15. Intervention. NCo. 2004.
16. Bang H, Ni L, Davis CE. Assessment of blinding in clinical trials. Control Clin Trials 2004; 25: 143-156.
17. Lancaster GA, Dodd S, Williamson PR. Design and analysis of pilot studies: recommendations for good practice. J Eval Clin Pract 2004; 10: 307-312.
18. Binda GA, Karas JR, Serventi A et al. Primary anastomosis vs nonrestorative resection for perforated diverticulitis with peritonitis: a prematurely terminated randomized controlled trial. Colorectal Dis 2012; 14: 1403-1410.
19. Oberkofler CE, Rickenbacher A, Raptis DA et al. A multicenter randomized clinical trial of primary anastomosis or Hartmann's procedure for perforated left colonic diverticulitis with purulent or fecal peritonitis. Ann Surg 2012; 256: 819-826; discussion 826-817.
20. Vennix S, Musters GD, Mulder IM et al. Laparoscopic peritoneal lavage or sigmoidectomy for perforated diverticulitis with purulent peritonitis: a multicentre, parallel-group, randomised, open-label trial. Lancet 2015; 386: 1269-1277.

For peer review only

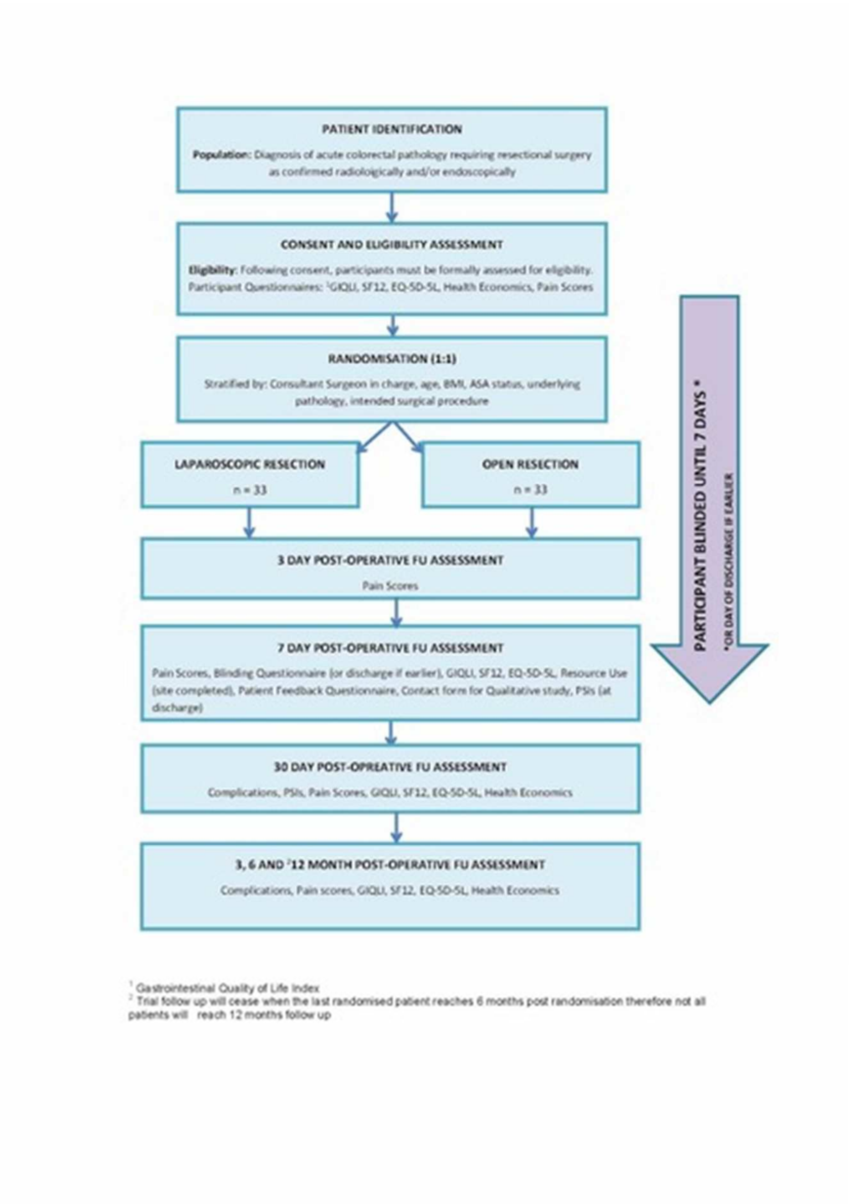

38x54mm (300 x 300 DPI)

STANDARD PROTOCOL ITEMS: RECOMMENDATIONS FOR INTERVENTIONAL TRIALS

SPIRIT 2013 Checklist: Recommended items to address in a clinical trial protocol and related documents*

Section/item	Page	Item No	Description
**Administrative information**			
Title	x	1	Descriptive title identifying the study design, population, interventions, and, if applicable, trial acronym
Trial registration	x	2a	Trial identifier and registry name. If not yet registered, name of intended registry
		2b	All items from the World Health Organization Trial Registration Data Set
Protocol version		3	Date and version identifier
Funding	X	4	Sources and types of financial, material, and other support
Roles and responsibilities	1-2	5a	Names, affiliations, and roles of protocol contributors
	x	5b	Name and contact information for the trial sponsor
		5c	Role of study sponsor and funders, if any, in study design; collection, management, analysis, and interpretation of data; writing of the report; and the decision to submit the report for publication, including whether they will have ultimate authority over any of these activities
		5d	Composition, roles, and responsibilities of the coordinating centre, steering committee, endpoint adjudication committee, data management team, and other individuals or groups overseeing the trial, if applicable (see Item 21a for data monitoring committee)
**Introduction**			
Background and rationale	4	6a	Description of research question and justification for undertaking the trial, including summary of relevant studies (published and unpublished) examining benefits and harms for each intervention
	4-5	6b	Explanation for choice of comparators
Objectives	5	7	Specific objectives or hypotheses
Trial design	5	8	Description of trial design including type of trial (eg, parallel group, crossover, factorial, single group), allocation ratio, and framework (eg, superiority, equivalence, noninferiority, exploratory)

For peer review only

Methods: Participants, interventions, and outcomes

Study setting	6	9	Description of study settings (eg, community clinic, academic hospital) and list of countries where data will be collected. Reference to where list of study sites can be obtained
Eligibility criteria	6	10	Inclusion and exclusion criteria for participants. If applicable, eligibility criteria for study centres and individuals who will perform the interventions (eg, surgeons, psychotherapists)
Interventions	8	11a	Interventions for each group with sufficient detail to allow replication, including how and when they will be administered
		11b	Criteria for discontinuing or modifying allocated interventions for a given trial participant (eg, drug dose change in response to harms, participant request, or improving/worsening disease)
		11c	Strategies to improve adherence to intervention protocols, and any procedures for monitoring adherence (eg, drug tablet return, laboratory tests)
		11d	Relevant concomitant care and interventions that are permitted or prohibited during the trial
Outcomes	9 - 10	12	Primary, secondary, and other outcomes, including the specific measurement variable (eg, systolic blood pressure), analysis metric (eg, change from baseline, final value, time to event), method of aggregation (eg, median, proportion), and time point for each outcome. Explanation of the clinical relevance of chosen efficacy and harm outcomes is strongly recommended
Participant timeline	10	13	Time schedule of enrolment, interventions (including any run-ins and washouts), assessments, and visits for participants. A schematic diagram is highly recommended (see Figure)
Sample size	11	14	Estimated number of participants needed to achieve study objectives and how it was determined, including clinical and statistical assumptions supporting any sample size calculations
Recruitment	7 and 10	15	Strategies for achieving adequate participant enrolment to reach target sample size

Methods: Assignment of interventions (for controlled trials)

Allocation:			
Sequence generation	7	16a	Method of generating the allocation sequence (eg, computer-generated random numbers), and list of any factors for stratification. To reduce predictability of a random sequence, details of any planned restriction (eg, blocking) should be provided in a separate document that is unavailable to those who enrol participants or assign interventions

Allocation concealment mechanism		16b	Mechanism of implementing the allocation sequence (eg, central telephone; sequentially numbered, opaque, sealed envelopes), describing any steps to conceal the sequence until interventions are assigned
Implementation		16c	Who will generate the allocation sequence, who will enrol participants, and who will assign participants to interventions
Blinding (masking)	8	17a	Who will be blinded after assignment to interventions (eg, trial participants, care providers, outcome assessors, data analysts), and how
		17b	If blinded, circumstances under which unblinding is permissible, and procedure for revealing a participant's allocated intervention during the trial
**Methods: Data collection, management, and analysis**			
Data collection methods	9 and 10	18a	Plans for assessment and collection of outcome, baseline, and other trial data, including any related processes to promote data quality (eg, duplicate measurements, training of assessors) and a description of study instruments (eg, questionnaires, laboratory tests) along with their reliability and validity, if known. Reference to where data collection forms can be found, if not in the protocol
	12	18b	Plans to promote participant retention and complete follow-up, including list of any outcome data to be collected for participants who discontinue or deviate from intervention protocols
Data management		19	Plans for data entry, coding, security, and storage, including any related processes to promote data quality (eg, double data entry; range checks for data values). Reference to where details of data management procedures can be found, if not in the protocol
Statistical methods	11	20a	Statistical methods for analysing primary and secondary outcomes. Reference to where other details of the statistical analysis plan can be found, if not in the protocol
		20b	Methods for any additional analyses (eg, subgroup and adjusted analyses)
		20c	Definition of analysis population relating to protocol non-adherence (eg, as randomised analysis), and any statistical methods to handle missing data (eg, multiple imputation)
**Methods: Monitoring**			
Data monitoring		21a	Composition of data monitoring committee (DMC); summary of its role and reporting structure; statement of whether it is independent from the sponsor and competing interests; and reference to where further details about its charter can be found, if not in the protocol. Alternatively, an explanation of why a DMC is not needed

		21b	Description of any interim analyses and stopping guidelines, including who will have access to these interim results and make the final decision to terminate the trial
Harms		22	Plans for collecting, assessing, reporting, and managing solicited and spontaneously reported adverse events and other unintended effects of trial interventions or trial conduct
Auditing		23	Frequency and procedures for auditing trial conduct, if any, and whether the process will be independent from investigators and the sponsor
**Ethics and dissemination**			
Research ethics approval	11	24	Plans for seeking research ethics committee/institutional review board (REC/IRB) approval
Protocol amendments		25	Plans for communicating important protocol modifications (eg, changes to eligibility criteria, outcomes, analyses) to relevant parties (eg, investigators, REC/IRBs, trial participants, trial registries, journals, regulators)
Consent or assent	7/8	26a	Who will obtain informed consent or assent from potential trial participants or authorised surrogates, and how (see Item 32)
		26b	Additional consent provisions for collection and use of participant data and biological specimens in ancillary studies, if applicable
Confidentiality		27	How personal information about potential and enrolled participants will be collected, shared, and maintained in order to protect confidentiality before, during, and after the trial
Declaration of interests	12	28	Financial and other competing interests for principal investigators for the overall trial and each study site
Access to data		29	Statement of who will have access to the final trial dataset, and disclosure of contractual agreements that limit such access for investigators
Ancillary and post-trial care		30	Provisions, if any, for ancillary and post-trial care, and for compensation to those who suffer harm from trial participation
Dissemination policy	12	31a	Plans for investigators and sponsor to communicate trial results to participants, healthcare professionals, the public, and other relevant groups (eg, via publication, reporting in results databases, or other data sharing arrangements), including any publication restrictions
		31b	Authorship eligibility guidelines and any intended use of professional writers
		31c	Plans, if any, for granting public access to the full protocol, participant-level dataset, and statistical code

For peer review only - http://bmjopen.bmj.com/site/about/guidelines.xhtml

Appendices

Informed consent materials	32	Model consent form and other related documentation given to participants and authorised surrogates
Biological specimens	33	Plans for collection, laboratory evaluation, and storage of biological specimens for genetic or molecular analysis in the current trial and for future use in ancillary studies, if applicable

*It is strongly recommended that this checklist be read in conjunction with the SPIRIT 2013 Explanation & Elaboration for important clarification on the items. Amendments to the protocol should be tracked and dated. The SPIRIT checklist is copyrighted by the SPIRIT Group under the Creative Commons "Attribution-NonCommercial-NoDerivs 3.0 Unported" license.

For peer review only
