# Supplementary material for: Feasibility of a multicentre, randomised controlled trial of laparoscopic versus open colorectal surgery in the acute setting: the LaCeS feasibility trial protocol
Source: BMJ Open. 2018 Feb 22;8(2):e018618. doi: 10.1136/bmjopen-2017-018618 (PMC5879497; doi:10.1136/bmjopen-2017-018618)
Supplement: Reviewer comments [file bmjopen-2017-018618.reviewer_comments.pdf]

PEER REVIEW HISTORY

BMJ Open publishes all reviews undertaken for accepted manuscripts. Reviewers are asked to complete a checklist review form (**http://bmjopen.bmj.com/site/about/resources/checklist.pdf**) and are provided with free text boxes to elaborate on their assessment. These free text comments are reproduced below.

ARTICLE DETAILS

**TITLE (PROVISIONAL)**	The feasibility of a multi-centre, randomised controlled trial of laparoscopic versus open colorectal surgery in the acute setting – The LaCeS Feasibility Trial Protocol
**AUTHORS**	Harji, Deena; Marshall, Helen; Gordon, Kathryn; Crow, Hannah; Hiley, Victoria; Burke, Dermot; Griffiths, Ben; Moriarty, Catherine; Twiddy, Maureen; O'Dwyer, John; Verjee, Azmina; Brown, Julia; Sagar, Peter

VERSION 1 – REVIEW

**REVIEWER**	Austin Acheson<br>University of Nottingham<br>Nottingham<br>UK<br>I am a member of the trial steering group for the laces study but i have no competing interests.
**REVIEW RETURNED**	17-Jul-2017

**GENERAL COMMENTS**	This is a very important feasibility study and I hope it leads to a large phase 3 RCT that will help to answer many questions on the area of emergency lap colorectal surgery. A very well written protocol that I would recommend publishing.

**REVIEWER**	Mr Valerio Celentano MD FRCS<br>Portsmouth Hospitals NHS Trust<br>University of Portsmouth<br>Portsmouth, United Kingdom<br>No conflict of interest
**REVIEW RETURNED**	28-Aug-2017

**GENERAL COMMENTS**	The trial aims to assess the feasibility, safety and acceptability of performing a large-scale definitive phase III randomised controlled trial comparing emergency<br>laparoscopic with open surgery for acute colorectal pathology.<br><br>There is increasing evidence supporting the use of laparoscopic surgery in the emergency setting, however this mainly consists of a number of retrospective case series and cohort studies. The trial aims to anwer the relevant question of the role of emergency laparoscopic colorectal surgery by recruiting 66 patients across five hospitals.

	Emergency surgery is currently undergoing a process of reconfiguration. Many services are still running a "general surgery" on call rota, where colorectal, oesophagogastric and hepatobiliary surgeons provide cross cover for all patients admitted with an acute general surgery pathology.<br>On the other hand, many hospitals have separate surgical teams covering an acute sub-specialty service, with often input from other subspecialty teams (i.e. GI radiology).<br>Nevertheless, the high costs and the impact on elective services of these models is yet to be fully investigated, therefore its applicability on a larger scale is debated.<br><br>For these reasons, it would extremely important to better define the configuration of the emergency general surgery service of the hospitals eligible to participate in the trial, with particular reference to availability of a dedicated "on call" colorectal surgeon. Moreover, it should be highlighted the need for a gastrointestinal radiology unit and the availability of an interventional radiologist for stenting. It should be mentioned if these services are also available out-of hours and during the weekend.<br><br>There is centralization of elective services, should it be the same for emergency colorectal resections?

**REVIEWER**	RICHARD GUY<br>DEPT OF COLORECTAL SURGERY<br>OXFORD UNIVERSITY HOSPITALS<br>CHURCHILL HOSPITAL<br>OXFORD, UK
**REVIEW RETURNED**	31-Aug-2017

**GENERAL COMMENTS**	This trial aims to address primarily the issue of recruitment to trials of emergency surgery, a perception that has been present for far too long. The nature of emergency abdominal surgery is that it doesn't readily lend itself to randomisation. For this reason alone, this trial is novel and should be supported, as the wealth of research opportunities in emergency surgery is hard to ignore, and the poor overall outcomes for emergency abdominal surgery need to be addressed.<br><br>My only slight concern with randomisation between open and laparoscopic surgery is the implication that these two approaches are mutually exclusive, although I am reassured by the seniority of the authors that this would not be their intention. However, I think this should be made clear in the manuscript. The intention to initially explore a patient laparoscopically is a good one, even if the procedure itself is not completed laparoscopically. For example, in a case of acute sigmoid diverticular perforation, it may be an advantage to mobilise the splenic flexure laparoscopically and therefore allow for a shorter lower midline incision to complete the resection. Similarly, mobilising the hepatic flexure for difficult acute terminal ileal Crohn's may be worthwhile. Such cases ought to be considered in the laparoscopic arm on an "intention to treat" basis. This is good practice, and it is preferred that the laparoscopic approach is simply another tool in the armamentarium of the emergency surgeon rather than a method to be used to the bitter end at all costs.

	I would like to see this made clear in the trial protocol and should in no way detract from the ability to recruit and randomise - indeed, put in this way it may be more acceptable to patients considering participation.

VERSION 1 – AUTHOR RESPONSE

We would like to thank the reviewers for their positive comments and for accepting our paper for publication.

We would like to respond to the following comments:

We have incorporated our surgeon eligibility criteria into the protocol paper to clarify what the meaning of the 'on-call' colorectal surgeon is. We have also included our site eligibility criteria to help clarify the configuration of the emergency general surgery services of the hospitals eligible to participate.

Laces is designed as a pragmatic trial and therefore we have not stipulated the need for a dedicated gastrointestinal radiology unit or the availability of an interventional radiologist for stenting. Furthermore, our protocol stipulates that patients are only to be approached to consider participation between 08:00 and 22:00, therefore circumventing the potential requirement of 24 hour radiology. In addition to this patients who are stented are not eligible to participate in the trial as per the eligibility criteria.

Conversion rates in the laparoscopic arm will be calculated in this trial. All analyses will be conducted on an intention to treat basis. The main aim of this trial is to assess the feasibility of recruiting and randomising patients in the emergency setting for acute colorectal pathology. We will be unable to draw any definitive conclusions about the laparoscopic-assisted or converted group until a definitive, phase III trial is conducted.

VERSION 2 – REVIEW

**REVIEWER**	Mr Valerio Celentano MD FRCS<br>Portsmouth Hospital NHS Trust, Portsmouth, United Kingdom<br>University of Portsmouth, Portsmouth, United Kingdom
**REVIEW RETURNED**	20-Oct-2017

**GENERAL COMMENTS**	All comments have been addressed

**REVIEWER**	RICHARD GUY<br>OXFORD UNIVERSITY HOSPITALS<br>UK
**REVIEW RETURNED**	15-Oct-2017

**GENERAL COMMENTS**	The authors have satisfactorily addressed the previously posed questions and concerns
